# Improved prediction of breast cancer risk based on phenotypic DNA damage repair capacity in peripheral blood B cells

**DOI:** 10.21203/rs.3.rs-3093360/v1

**Published:** 2023-06-27

**Authors:** Hazeem L. Okunola, Igor Shuryak, Mikhail Repin, Hui-Chen Wu, Regina M. Santella, Mary Beth Terry, Helen C. Turner, David J. Brenner

**Affiliations:** Columbia University Irving Medical Center; Columbia University Irving Medical Center; Columbia University Irving Medical Center; Columbia University Mailman School of Public Health; Columbia University Mailman School of Public Health; Columbia University Mailman School of Public Health; Columbia University Irving Medical Center; Columbia University Irving Medical Center

**Keywords:** Breast cancer prediction, Phenotypic assay, DNA repair capacity

## Abstract

**Background:**

Standard Breast Cancer (BC) risk prediction models based only on epidemiologic factors generally have quite poor performance, and there have been a number of risk scores proposed to improve them, such as AI-based mammographic information, polygenic risk scores and pathogenic variants. Even with these additions BC risk prediction performance is still at best moderate. In that decreased DNA repair capacity (DRC) is a major risk factor for development of cancer, we investigated the potential to improve BC risk prediction models by including a measured phenotypic DRC assay:

**Methods:**

Using blood samples from the Breast Cancer Family Registry we assessed the performance of phenotypic markers of DRC in 46 matched pairs of individuals, one from each pair with BC (with blood drawn before BC diagnosis) and the other from controls matched by age and time since blood draw. We assessed DRC in thawed cryopreserved peripheral blood mononuclear cells (PBMCs) by measuring γ-H2AX yields (a marker for DNA double-strand breaks) at multiple times from 1 to 20 hrs after a radiation challenge. The studies were performed using surface markers to discriminate between different PBMC subtypes.

**Results:**

The parameter $F_{\text{res}}$, the residual damage signal in PBMC B cells at 20 hrs post challenge, was the strongest predictor of breast cancer with an AUC (Area Under receiver-operator Curve) of 0.89 [95% Confidence Interval: 0.84–0.93] and a BC status prediction accuracy of 0.80. To illustrate the combined use of a phenotypic predictor with standard BC predictors, we combined $F_{\text{res}}$ in B cells with age at blood draw, and found that the combination resulted in significantly greater BC predictive power (AUC of 0.97 [95% CI: 0.94–0.99]), an increase of 13 percentage points over age alone.

**Conclusions:**

If replicated in larger studies, these results suggest that inclusion of a fingerstick-based phenotypic DRC blood test has the potential to markedly improve BC risk prediction.

## Introduction

Breast cancer (BC) is the second leading cause of cancer deaths in women worldwide [1–3], and is an increasing public health problem [4–7]. Global BC cases are increasing by more than 3% annually with over one third of world BC cases currently diagnosed under the age of 50 [4, 8–11]. The rate of BC is also increasing in the US, especially for women under the age of 40 [12, 13], and it is the leading cause of death for women in their 40s [1–3].

The high and increasing rate of BC cases worldwide emphasizes the need for sensitive and specific BC risk assessment tools. Standard questionnaire-based BC risk prediction models generally have quite poor performance with AUCs (Area Under receiver-operator Curve) rarely above 0.65 [14] for general population cohorts. Consequently there have been a number of factors proposed to augment and improve them, such as AI-based mammographic information, polygenic risk scores and pathogenic variants [15–20]; however, even with these additions, current BC risk prediction is still, at best moderate, with AUCs rarely above 0.7 [15–20].

This modest performance makes it challenging to target effective primary prevention options (*e.g*., chemoprevention) for the great majority of women who are not known to have pathogenic variants in breast cancer susceptibility. Further, the efficiency of secondary prevention options (*e.g.*, onset, frequency, and method of BC screening by mammography or other supplemental methods) would also benefit from more accurate BC risk prediction. Based on these consideration, a desirable target for breast cancer screening performance in the context of screening, counseling, prevention would be an AUC of at least 0.8 [14], and thus the current study is designed to investigate a different class of BC predictor which may be combined with present models to further improve BC predictor performance.

In this context DNA damaging agents such as smoking, ionizing radiation, exogenous estrogens, alcohol intake and diets that increase the risk of obesity [21–27] have all been associated with breast cancer risk. When comparing tumors with nearby non-tumor tissues, breast tumors have significantly more DNA damage [28–32] and DNA adducts [28, 29, 33, 34]. For these reasons, we and other groups have been assessing predictive biomarkers that could enhance BC risk prediction: several reports, including those from our own team [35–37], have suggested that decreased DNA repair capacity (DRC) is a major risk factor for development of cancer at many sites including lung, bladder, as well as breast [38]. The goal of the present study is to develop this approach with the goal of improving personalized BC predictive models.

DNA repair is a crucial mechanism for maintaining genomic stability in cells. Defects in the DNA repair machinery increase cell vulnerability to DNA-damaging agents and accumulation of mutations in the genome, and lead to the development of various disorders including cancer. Studies that have measured DRC, including our own, have estimated that DRC is associated with a much higher risk of BC (3–15-fold) [37], than most other established BC risk factors, with the exception of highly penetrant mutations in genes like *BRCA1* and *BRCA2*, genes that are themselves critical to DNA repair. This lack of inclusion of a major risk factor – DRC – may be one of the reasons that current clinical BC risk models have only modest performance.

DRC can be assessed with genotypic, proteomic, or phenotypic approaches. A concern with genomic or proteomic approaches is that mammalian DNA damage repair mechanisms are extraordinarily complex, in humans involving ~ 450 genes, 13 different pathways, with over half the proteins interacting with other proteins from different pathways [39]. Most BC risk models now try to capture genetic risk variants in DNA repair but that genotype typically only explains a small portion of the variation in phenotype [40–44]. Consequently, any specific genomic or proteomic methodology may not reflect overall DRC.

By contrast to genotypic and proteomic approaches, phenotypic approaches such as inducing DNA damage and then measuring the rate of DNA damage repair or the amount of unrepaired/misrepaired DNA damage, or both, have the potential to be more reflective of overall DRC. To date, however, such phenotypic approaches have been laborious to perform resulting in low throughput, and there have been no large-scale prospective studies of the relationship between BC and DRC. In the current study, we have overcome this by using an automated phenotypic approach to DRC characterization, based on measuring the kinetics of DNA double-strand break (DSB) damage repair [45–47] after a radiation challenge.

Specifically, measurement of phosphorylation of histone H2AX at Ser 139 at the site of DNA DSB has become one of the most common DSB assays [48–50] and here we measure the repair of γ-ray induced DNA DSB over time by observing the disappearance of γ-H2AX signals using an image-flow cytometer [45–47]. Of course PBMCs consist of a number of different cell subtypes and it is known that DSB induction sensitivity differs in different PBMC subtypes [51], and thus their DRCs may also vary. For this reason we separately analyzed the different PBMC subtypes, identified using standard surface markers. Using the automated high-throughput RABiT (Rapid Automated Biodosimetry Technology) approach [45, 52–60], time-dependent measurements of the γ-H2AX DSB biomarker after a radiation challenge, together with use of surface markers for different PBMC cell types and a multi-channel image-flow cytometer, allowed us to directly quantitate DRC in each PBMC subtype.

We quantitatively characterized DRC in blood samples from 92 women, 46 of whom were diagnosed with BC, and 46 matched healthy controls. For the women with BC, the blood samples were drawn and stored *before* BC diagnosis, and the controls were matched by age and time since blood draw. Our goal was to investigate whether parameters derived from the blood sample DRC measurements could be predictive of the risk of BC diagnosis.

## Methods

### The Breast Cancer Prospective Family Study Registry

The Breast Cancer Prospective Family Study Registry (BCFR) [61] is a prospective cohort of 31,640 women from 11,171 families ascertained through population-based and clinic-based sampling, covering the full spectrum of familial risk. Recruitment commenced in 1992 from families in the USA, Canada and Australia. Blood samples (30 ml) have been collected from 83% of the cohort, and processed for storage with a standardized protocol. Multi-generational cancer family histories were sought from all participants who completed the same structured epidemiologic questionnaire, obtaining information on demographics, race/ethnicity, personal history of cancer, breast and ovarian surgeries, radiation exposure, smoking and alcohol consumption, menstrual and pregnancy history, hormone use, weight, height, physical activity and medical diagnostic radiation. As part of the ongoing follow-up, first- and second- degree family history of cancer continues to be updated for all participants. The cohort includes blood samples from approximately 10,000 women who have a primary invasive breast cancer and from than 20,000 women who do not have breast cancer.

For this current study we selected samples using a nested case-control study design, using blood samples that were collected blood prior to breast cancer diagnosis and matching controls based on time of followup and age at baseline blood draw. The 92 frozen PBMC samples used in the current study (46 prospective breast cancer cases and 46 matched controls) were accessed from the New York site of the Breast Cancer Prospective Family Study Registry [61].

### The RABiT: High throughput technology for assessing global DRC

The RABiT (Rapid Automated Biodosimetry Tool) was developed at Columbia University in the context of providing a high-throughput biodosimetry assay after a large-scale radiological event [57–59]. It was subsequently adapted for high-throughput global DRC quantification measuring the time dependence after a DNA damage challenge of repair proteins such as γ-H2AX, 53-BP1, ATM kinase and MDC1 [45–47]. The RABiT is an automated high-throughput robotically-based tool which is designed to take as input fingerstick volumes (~ 50 μl) of blood. The design of the global DRC methodology involves inducing DNA damage to the PBMC, here with gamma rays, followed by repeated automated sampling of the cells as a function of time after the radiation challenge. In this study, the γ-H2AX assay, a measure of DNA DSB, was used.

### Thawing frozen PBMCs

Cryopreserved PBMCs were thawed rapidly in a 37°C water bath and transferred to sterile, 15 mL Bio-Reaction tubes with ventilated screw caps with 0.22 μm filters (CellTreat, Pepperell, MA). MarrowMAX^™^ Bone Marrow Medium (ThermoFisher) was added dropwise to the thawed cells using sterile transfer pipettes to a volume of 10 mL and gently mixed by pipetting up and down with a 1 ml pipet tip to ensure a homogeneous solution. We centrifuged cells for 10 min at 300-xg in a hanging bucket rotor centrifuge (ThermoScientific Sorvall LEGEND XT Centrifuge) at room temperature. After centrifugation, we removed the supernatant without disturbing the pellet, resuspended the pellet in 10 mL of MarrowMAX medium and centrifuged again at 300-xg for 10 min. After the second centrifugation, we again resuspended the pellet in 10 mL of MarrowMAX medium and incubated it in a humidified 37°C incubator with 5% CO_2_ for 1 hr. After incubation, we centrifuged the cells again at 300-xg for 10 min, removed supernatant and resuspended the pellet in 10 mL Gibco RPMI 1640 Medium (Fisher Scientific) with 15% Gibco Fetal Bovine Serum (ThermoFisher), qualified and heat inactivated with 1% Penicillin-Streptomycin (10,000 U/mL) (ThermoFisher). After cell resuspension, we took an aliquot of 18 μL from each donor sample and mixed it with 2 μL of Acridine Orange/Propidium Iodide stain (AO/PI) (Logos Bio, Annandale, VA). We then transferred 20 μL of stained cells into a photon slide (Logos Bio) and counted the cells with a LUNA dual fluorescence automated cell counter (Logos Bio), and recorded the numbers of live cells and cell viability.

### PBMC irradiations

We divided the 10mL culture into prelabeled 1.2 ml sterile 2D Barcoded, V Bottom Screw-Top Matrix Tubes w/ Caps (ThermoFisher). Two unirradiated controls, and two tubes for channel compensation were also included. We exposed the tubes to a 3-Gy gamma-ray dose using a Gammacell-40 ^137^Cs irradiator (Atomic Energy of Canada, Chalk River, ON, calibrated using an Optically-Stimulated Luminescence dosimeter). After irradiation, we immediately incubated the samples without the caps in a humidified 37°C incubator with 5% CO_2_. At the appropriate post-irradiation time points (1, 2, 3, 6, or 20 hrs) we removed the tubes, processed, permeabilized and fixed the cells with BD Cytofix/Cytoperm buffer (BD Scientific) for 20 min at 4°C in the dark.

### Surface markers for PBMC subtypes and γ-H2AX immunolabeling/phenotyping assay

Use of the ImageStream^X^ Mk II imaging flow cytometer (Luminex Corporation, Austin) with 12 channels and 5 different excitation lasers, enabled a multi-chromophore γ-H2AX phenotyping assay that used 8 different chromophores in the same reaction tube at the same time. An antibody cocktail which included the conjugated antibody for γ-H2AX-FITC and the surface antibodies CD3^+^-Alexa-Fluor647, CD4^+^-PE, CD8a^+^-PerCP/Cy5.5, TCRγδ^+^-BV510, CD19^+^-APC/Cy7, CD56^+^-PE/Dazzle594 and CD14^+^-BV421 was prepared with appropriate dilutions and added to the fixed cells in the matrix tubes. Details and sources of the antibodies are listed in the Supplementary Information.

We incubated the homogeneous cell-mixture at 4°C overnight. The unbound antibodies were washed twice with 1X BD perm/wash buffer (BD Scientific), followed by two washes of 1X DPBS (Fisher Scientific). We centrifuged the samples in a hanging plate adapter rotor centrifuge (Sorvall LEGEND XT Centrifuge, ThermoScientific) for 3 min at 300-xg after every wash at room temperature. After the last 1X DPBS wash, most of the supernatant was removed from the matrix tube and the ~ 50 μL supernatant left in the tube was used to resuspend the pellet and transfer samples to 1.5 mL Eppendorf tubes which were stored at 4°C for Image Flow analysis.

### Data acquisition and analysis

We transferred the samples to the flow cytometer for manual sample acquisition and image capturing using the ISX INSPIRE^™^ data acquisition software. We acquired 1300 images of single cells per sample at 40X magnification using the 375, 405, 488, 561 and 642 nm excitation lasers at 200 mW. We captured in separate channels bright field (BF) images, γ-H2AX immunostaining, CD3^+^, CD4^+^ CD8a^+^, CD14^+^ CD19^+^, CD56^+^, TCRγδ^+^, and other BF images. At the end of data acquisition, we compensated for any fluorescence spillover emission using IDEAS 6.3 software (Luminex Corporation) as described elsewhere [62].

### Quantitative modeling of DNA damage repair kinetics

The data were normalized by subtracting the 2 hr 0-Gy measurements from the 1 2, 3, and 6 hr 3-Gy time points, and subtracting the 20 hr 0-Gy measurements from the 20 hr 3-Gy data. These normalizations allowed us to correct for potential drift in the 0-Gy control data.

We modeled the time course of the signal (Y) for each cell subtype after irradiation separately for each donor using the equation below, where D is radiation dose (Gy), T is time (hrs) after irradiation, Ω is a binary indicator variable for irradiation (Ω=1 for D>0,Ω=0 for D=0). $K_{\text{prod}}$ is the rate of signal production after radiation, $K_{dec}$ is the initial exponential signal decay (repair) rate, $F_{res}$ is the residual signal (unrepaired/misrepaired DSB) after overnight incubation:

(1) $$Y=\left[K_{\mathrm{prod}}\times D\times T\times exp\left(-K_{dec}\times T\right)+F_{\mathrm{res}}\times D\right]\times \Omega .$$

We fitted the model (Eq. 1) using a customized least squares procedure written in Maple 2021 software. The parameter $F_{res}$ was estimated based on the observed signal value at 20 hrs (i.e. $F_{\mathrm{res}}\times D$ was assumed to equal the observed signal at 20 hrs), and $K_{\mathrm{prod}}$, and $K_{dec}$ were fitted by least squares on a natural log scale using all time points. Since the radiation dose D was fixed in our experiments, for convenience we abbreviate the expression $F_{\mathrm{res}}\times D$ simply as $F_{res}$ throughout the analysis description below, because only the correlation with breast cancer status (and not the absolute units of $F_{res}$) is important for our analysis.

### Potential predictors of breast cancer status

We classified the samples by BC status using the Breiman random forest (RF) machine-learning algorithm [63], implemented using the *ranger* package in *R* 4.2.0 software. This RF algorithm generates many uncorrelated decision trees by bootstrap aggregation (randomly selecting samples from training data with replacement) and feature randomness (selecting a random subset of predictor variables for each tree). The algorithm then averages the predictions from all trees.

The potential predictor variables used in our RF model were the estimated $F_{res}$ and $K_{\mathrm{dec}}$ parameters for each studied cell subtype. In addition, as a first investigation of the potential for combining “standard” BC predictors with phenotypic parameters we investigated adding the demographic variable of “age at blood draw” as an additional predictor of BC status.

The initial feature selection step, intended to detect the strongest predictors of BC status among the various candidates listed above, was performed using the Boruta algorithm [64]. This algorithm iteratively compares the importance score of each predictor with the importance score of its randomly shuffled “shadow”, in the context of a random forest model [63]. It duplicates the data set and randomly shuffles the values in each column. These shuffled “shadow features” are re-created in each iteration. We discarded those predictors that had significantly (p<0.05 with Bonferroni correction) worse importance scores than the shadow features.

## Results

**Figure 1.**
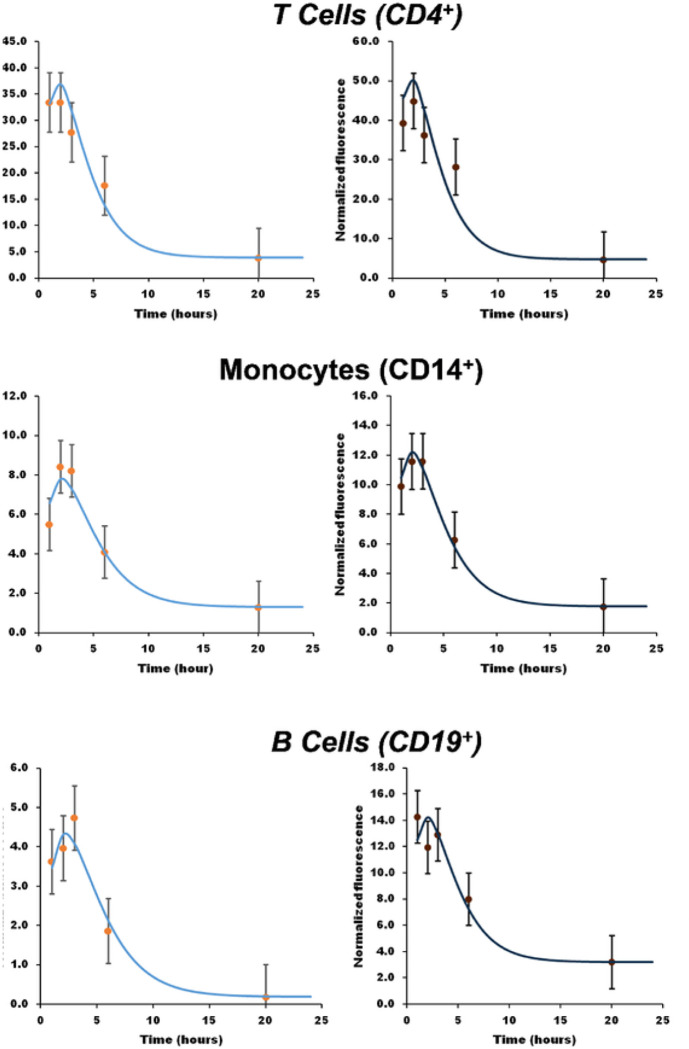
Illustrating typical time-dependent g-H2AX fluorescence measurements for three of the measured cell sub-types in a matched pair of blood samples (Left: Breast cancer case [blood drawn before BC diagnosis]; Right: matched healthy control). Points: Experimental measurements. Curves: Model fits to the data using Eq. (1).

Complete results, including all the measured time-dependent data and the corresponding model-fit parameters ($K_{\mathrm{dec}},\;F_{res}$, see Eq. 1) are shown in the Supplementary Information for each of the 92 donors (46 BC and 46 matched controls) and for each cell subtype. Illustrated in Fig. 1 are examples of results and model fits for a pair of matched donors (BC and non-BC) for 3 cell sub-types.

### Assessing DRC parameters as potential predictors of breast cancer status

**Figure 2.**
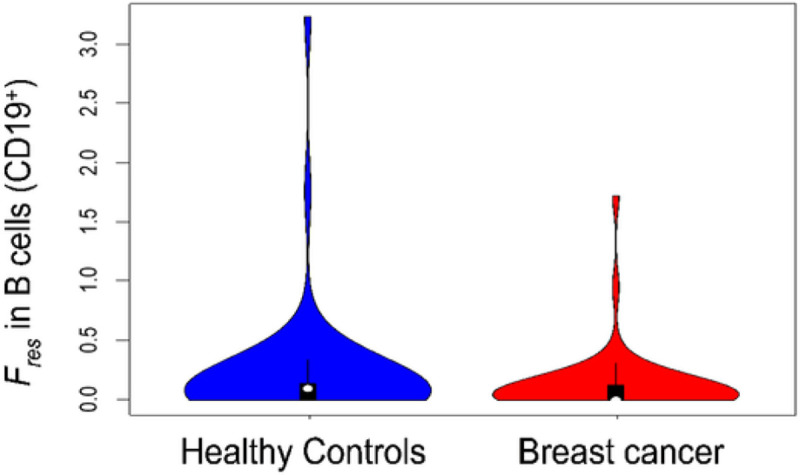
*Violin plots for* $F_{res}$ *in B cells (CD19*^*+*^), illustrating the results for BC cases (n=46) and matched healthy controls (n=46). A U-test comparing the distributions yielded a p-value of 0.12.

We focused on parameters $K_{\mathrm{dec}}$ and $F_{res}$ as potential predictors of breast cancer status and used Boruta and Random Forest (RF) algorithms to identify the strongest predictors for BC status and to build a classification model using these predictors. Out of all tested time course model parameters ($F_{res}$ and $K_{\mathrm{dec}}$ for CD3^+^, CD4^+^, CD8a^+^, CD14^+^, CD19^+^, CD56^+^, and TCRγδ^+^), only $F_{res}$ for CD19^+^ (B cells) was confirmed by the Boruta algorithm as a strong predictor of BC status, and all the others were rejected. We fitted an RF model to the data using this single variable (B-cell $F_{res}$) to predict BC status. Figure 2 shows a Violin plot of the distributions of the estimated B-cell $F_{res}$ values in the cases and the controls. The Spearman correlation coefficient for B-cell $F_{res}$ and BC status was r=-0.15(p=0.15). Of interest here, and as discussed below, is that BC status was correlated with decreased $F_{res}$ values.

**Figure 3.**
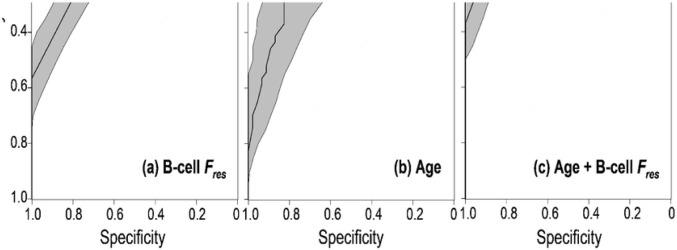
ROC curves for random forest models using different predictors of breast cancer status. Shading indicates the 95% CI region. **a:** Using just $F_{res}$ in B cells as the predictor: Area under the curve (AUC) is 0.89 [95% CI: 0.84–0.93], and prediction accuracy is 0.80 [95% CI: 0.71–0.88]. **b:** Using just Age as the predictor: AUC is 0.84 [95% CI: 0.76–0.92], and prediction accuracy is 0.76 [95% CI: 0.66–0.84]. **c:** Using both $F_{res}$ in B cells and Age as predictors: AUC is 0.97 [95% CI: 0.94–0.99], and prediction accuracy is 0.91 [95% CI: 0.84–0.96].

We fitted an RF model to the data using this single B-cell $F_{res}$ variable, and the resulting ROC curve is shown in Fig. 3a. The area under the ROC curve (AUC) for this model was 0.89 [95% Confidence Interval CI: 0.84–0.93], based on bootstrapping 10,000 times, and the corresponding BC prediction accuracy of this B-cell $F_{res}$ model was 0.80 [95% CI: 0.71–0.88]. These findings suggest that $F_{res}$ in B cells has encouraging predictive power for BC status in the analyzed data set.

As discussed above, we do not view phenotypic parameters as potential stand-alone predictors of breast cancer status, but rather as augmenting and potentially improving current BC risk prediction models. To illustrate combining the $F_{res}$ phenotypic predictor identified here with more commonly-used BC predictors, we investigated the effect of combining the B-cell $F_{res}$ predictor together with age (here age at blood draw). Age is typically the strongest demographic predictor in standard BC predictive models [65].

Considering age alone as a BC predictor in the data set studied here, the corresponding RF model ROC curve (Fig. 3b) AUC was 0.84 [95% CI: 0.76–0.91], with prediction accuracy of 0.76 [95% CI: 0.66–0.84]. Adding B-cell $F_{res}$ to age (Fig. 3c) significantly increased the BC prediction accuracy from 0.76 to 0.91 (p=0.0087, using Fisher’s exact test), and significantly increased the ROC curve AUC from 0.84 (for age alone) to 0.97 [95% CI: 0.94–0.99] (p=0.00049, using DeLong’s test [66]).

## Discussion

### Lower measured residual DNA damage in PBMC B cells from BC patients

The observation that B cells from BC patient PBMCs showed lower measured levels of residual damage ($F_{res}$) might at first appear counterintuitive. We had previously observed [35] a lower DNA repair capacity relative to controls in lymphoblastoid cell lines (LCL) derived from BC patients – although the relation between repair mechanisms in LCL and in PBMC is not well established [67]. In the current context the lower measured levels of residual damage in BC patient PBMC B cells may be associated with increased error-prone DNA repair [68–70], resulting in lower measured residual DNA damage but correspondingly increased levels of unmeasured misrepaired chromosome-level lesions. If mechanisms such as this were significant, the approach discussed here might not be specific to breast cancer, but could potentially be useful for predicting other common cancers

### Potential for augmenting current BC risk predictors with a phenotypic BC predictor

Our findings in a study with 48 BC cases (with blood drawn prior to BC diagnosis) and 48 matched controls suggest that residual γ-H2AX damage (designated here as $F_{res}$), measured in PBMC B cells at 20 hrs after DNA damage challenge, is systematically lower in women who were subsequently diagnosed with breast cancer, relative to the matched controls. If validated in larger studies, this suggests that such phenotypic measurements might form the basis for a potentially useful additional predictor of BC, when combined with currently-used BC predictors. As an example, we combined $F_{res}$ in B cells with the demographic predictor “age at blood-draw”, and found the combination resulted in significantly more BC predictive power than either on its own, as illustrated, for example, by a 13 percentage point increase in ROC curve AUC compared with age alone.

Measurements of residual DNA damage in PBMC B cells at ~ 20 hours after a DNA damage challenge would not be technologically challenging. Following the approach described here, only a fingerstick (30 μl) of blood would be needed, which would be given a radiation challenge followed by an immunohistochemical assay 20 to 24 hours later. Turner *et al* [45] have described a simple small fingerstick blood irradiator that could be used here.

## Conclusions

Despite contemporary improvements in breast cancer risk predictors, their performance is still quite moderate (AUC typically less than 0.7). However it is clear that AUCs of at least 0.8 would be highly advantageous from the perspectives of screening, counseling and prevention. In this context, we investigated here a new class of predictor, phenotypic DNA repair capacity (DRC), which could potentially be added to the current predictors. If replicated in larger studies, the results here suggest the possibility that a practical phenotypically-based DRC assay, when used in conjunction with current BC risk prediction models, may yield significantly improved BC predictive power as compared to what is currently achievable.
